# Fundamentals of safety hazards: A scientific perspective

**DOI:** 10.4102/jamba.v11i1.675

**Published:** 2019-06-18

**Authors:** Elriza Esterhuyzen, Leonie B. Louw

**Affiliations:** 1 Department of Operations Management, University of South Africa, Pretoria, South Africa

**Keywords:** safety risk, safety hazard characteristics, hazard identification, origin of safety risk, G20

## Abstract

Current theory on safety hazards and the origin of safety risk is often unstructured, misleading and ambiguous. Essentially, it is ambiguous, as definitions and descriptions refrain from stating a formal common basis upon which one can rely to fundamentally and rightfully conclude what a safety hazard is. As a result, it is quite an effort to set a scientifically valid base for precisely what safety hazards are. The objective of this study was to outline the questionable bases of current views on safety hazards and identify the real nature of safety hazards. The characteristics of safety hazards inform the verification of the scientific nature of the different perspectives on safety hazards. Through a quantitative survey, an assessment was performed regarding the cognisance of South African small business owners and managers related to safety hazards. This study found that safety hazards need to unambiguously remain safety hazards under all circumstances in life. Small business owners and managers require further education to develop their cognisance of safety hazards in order to manage the related safety risk. This research has indicated that not all small business owners or managers are compliant with this legal responsibility and that assistance should be provided to small business owners or managers to assist them in realising the importance of safety hazards in the workplace. Proper cognisance of safety hazards leads to enhanced compliance with legislative requirements.

## Introduction

The Group of Twenty (G20), as the forum for international cooperation, with South Africa constituting one of the member countries, indicates in its ‘Statement on Safer and Healthier Workplaces’ that the identification of safety hazards in the workplace is a core component of preventing injury and loss (G20 2017). Current theory on safety hazards and the origins of safety risk is often unstructured and misleading. Essentially, it is ambiguous, as definitions and descriptions refrain from stating a formal common basis upon which one can rely to fundamentally and rightfully conclude what a safety hazard is. As a result, it is quite an effort to set a scientifically valid base for precisely what safety hazards are. Postulations about what real safety hazards specifically constitute are confusing and do not present a solid framework that can lead to a clear, uniform and scientifically acceptable understanding of the real nature of safety hazards. Research has shown that even though South African small business owners or managers do recognise the importance of safety hazards in the workplace, their cognisance in this regard is not at the desired levels.

### Objectives

This article will address the questionable bases of current views on safety hazards, and explain the real nature of safety hazards. The characteristics of safety hazards will be investigated and it will verify the scientific nature of the different perspectives on safety hazards. It will further assess the cognisance of South African small business owners or managers related to safety hazards.

This article will consider most of the current views on and definitions of hazards. It will provide an overview of the confusing and misleading elements within these views and argue the solid scientific base. The argument will *ipso facto* indicate the invalidity or validity of the different views. Small business owners’ or managers’ cognisance of safety hazards in their businesses are also investigated and discussed.

## Research methodology

Primary and secondary data were utilised to gather the necessary information to meet the objectives stated above. Secondary data comprised a literature review to ascertain the current views on safety hazards. Primary data were gathered using a questionnaire (as measuring instrument) and statistically analysed. This quantitative study was conducted in the three South African provinces comprising the majority of active small businesses: Gauteng, KwaZulu-Natal and the Western Cape. For the purpose of this study, a small business comprised those with an annual turnover of less than R10 million and fewer than 50 employees. The owners or managers of 350 small businesses took part in this study (Esterhuyzen 2017).

## Current views on safety hazards

Current literature lists different definitions of the term ‘safety hazard’. Various options, as listed below, are offered as definitions of safety hazards, representing the current views on safety hazards.

### A condition or situation is a safety hazard

The view that a situation or condition is a safety hazard is shared by various authors, such as the National Safety Council (1996:750), Thygerson (1977:39), Bird and Germain (1996:28), Germain, Bird and Labuschagne (2011:544), Fuller and Vassie (2004:6), Goetsch (2005:294, 577), Germain et al. (1998:64), Roland and Mortiarty (1990:6), Terry (1991:55), Manuele (1993:26), NOSA (1994a:19), Keller and Associates (1999:27), Hansen (2012:68) and Vincoli (1997:12).

### A predisposition is a safety hazard

A disposition can, amongst others, be regarded as a tendency, inclination or attitude (Complete Wordfinder 1993). Fuller and Vassie list a predisposition as a safety hazard (2004:6). Allport, Gilbert and Outterside (2003) sees a disposition as a trait that inspires human behaviour.

### A method or process of work is a safety hazard

Quite a few authors regard a method or process of work as a safety hazard, for example, Stranks (2010:42), Bird and Germain (1996:28), Germain et al. (1998:64), Germain et al. (2011:544), as well as Keller and Associates (1999:27).

### A human act is a safety hazard

Human acts are listed as safety hazards by Fuller and Vassie (2004:28), Roland and Moriarty (1990:197) and Seabrook et al. (2012:40).

### A source of behavioural factors that cause harm is a safety hazard

This viewpoint regards sources that are left uncontrolled and lead to human harm as safety hazards. There is no specification between material and immaterial sources. The viewpoint is underscored by Grimaldi and Simonds (1989:181), Mroszczyk (2012:163) and Blayney (2012:925).

### Departure from the normal is a safety hazard

Stranks (2010:89) indicates that any departure from the normal presents a safety hazard.

### Exposure is a safety hazard

NOSA (1994b:197) sees exposure to safety risk as a safety hazard. *The Occupational Health and Safety Act* (*OHS Act*) *1993* (Section 1) also stipulates exposure as a safety hazard.

### Safety risk associated with loss or damage is a safety hazard

Cascarino and Van Eck (2007:43) argue that safety risk that leads to loss or damage to assets presents a safety hazard.

### Stress is a safety hazard

Geller (1998:60), Goetsch (2010:v), Stranks (2010:162) and Thygerson (1986:79) regard stress as a safety hazard.

### An event is a safety hazard

That an event is a safety hazard is an opinion that is shared by Hansen (2012:68) and Smith (2012:370).

### Tangible objects or substances are safety hazards

Van Fleet (2000:112) posits that a safety hazard is ‘any tangible object that has the potential to complement or interfere with the performance of a task’. Grimaldi and Simonds (1989:181) maintain that safety hazards are sources of energies that have the potential to damage, while Stranks (2010:42) contends that safety hazards, such as substances and machines, can cause damage. Stephenson (1991:8) and Fields (2012:64) are of the same opinion.

## Scientific qualities applicable to real safety hazards

For anything to be regarded as a safety hazard, a requirement is that specific scientific-based qualities have to be revealed. Such qualities are tangibility, movability, interactability and unambiguousness, which will be briefly discussed below.

### Tangibility or contactability (collision potential)

For any specific object or substance to have the potential to harm humans or the natural and the developed environment, a requirement is that such an object or substance must have the potential to contact other objects or substances. The ability to make contact relates to the fact that all substances are made of matter which manifests in the observable universe (Zumdahl & Zumdahl 2007:25). Matter constitutes the solidity of objectives or substances. The mass and space or volume of all objects or substances relates to the material basis of those objects or substances.

Thus, if there is no material (matter) base, there is no contactability. If an object or substance does not have the ability to make contact, it has no potential to cause harm or loss. Van Fleet (2000:112) contends that only tangible objects have the potential to impact life positively or adversely. Therefore, no contactability, no safety hazard and all objects or substances that have the quality of contactability are safety hazards. Van Fleet (2000:112) uses the term ‘collision potential’ to refer to the safety hazard quality of tangibility or contactability.

### Movability (closing potential)

If objects or substances are to make contact, they must have the potential to move on their own or be moved by other objects or substances (Van Fleet 2000:112). Anything that does not have the potential or quality of moving cannot be a safety hazard.

### Interactability

When objects or substances contact other objects or substances, they enter into some form of interaction with one another (Van Fleet 2000:112). When objects or substances interact, some form of energy exchange occurs. For example, a partially eaten apple gradually turns brown as a result of interacting with the oxygen in the air and the apple oxidises. The process and outcome, results or effects of interacting can be advantageous or adverse.

### Unambiguousness

If an object or substance is a safety hazard, it must remain a safety hazard always and under all conditions in life. The definition of a safety hazard must meet the scientific criterion of unambiguousness. The definition of what an object or substance is, or what object or substance constitutes a safety hazard, cannot allow, imply or provide for a double meaning. A safety hazard is a safety hazard, without the possibility of any argument about it. There is no chance for an object or substance to become or develop into a safety hazard, depending on the circumstances. A safety hazard does not have the ability to change from being a safety hazard now to not being a safety hazard at another time.

### Test for real safety hazards

The test to determine whether a thing, object or substance is a safety hazard lies in verifying to what extent such a substance meets the four qualifications as listed above, namely tangibility (collision potential), movability (closing potential), interactability and unambiguousness. Table 1 confirms the extent to which the safety hazards, as listed and defined above, are valid in meeting all four of these qualities.

**TABLE 1:** Validity of current views on safety hazards.

No.	Definition of safety hazard	Safety hazard qualities with confirmation of validity							
		Tangibility		Movability		Interactability		Unambiguousness	
		Yes	No	Yes	No	Yes	No	Yes	No
1	A condition or situation is a safety hazard	-	√	-	√	-	√	-	√
2	A predisposition is a safety hazard	-	√	-	√	-	√	-	√
3	A method or work process is a safety hazard	-	√	-	√	-	√	-	√
4	A human act is a safety hazard	-	√	-	√	-	√	-	√
5	A source of behavioural factors that cause harm is a safety hazard	-	√	-	√	-	√	-	√
6	Departure from the normal is a safety hazard	-	√	-	√	-	√	-	√
7	Exposure is a safety hazard	-	√	-	√	-	√	-	√
8	Safety risk associated with loss or damage is a safety hazard	-	√	-	√	-	√	-	√
9	Stress is a safety hazard	-	√	-	√	-	√	-	√
10	An event is a safety hazard	-	√	-	√	-	√	-	√
11	Tangible objects are safety hazards	√	-	√	-	√	-	√	-

The details in Table 1 clearly indicate that 10 of the 11 definitions of a safety hazard in the literature on safety do not meet any one of the basic qualities of real safety hazards. In this regard, it is quite clear that only substances that are made of matter and that are therefore tangible are safety hazards.

## Characteristics of safety hazards

Safety hazards have specific characteristics that make them dangerous. The danger that they offer or pose takes the form of safety risk. Substances that are safety hazards all carry the characteristics of safety hazards, which can be classified as structural and functioning characteristics, as discussed below.

### Structural characteristics of safety hazards

Because of their material (matter) base, all safety hazards have some form of structure that holds the safety hazard as a substance together and allows it to retain its shape (Zumdahl & Zumdahl 2007:25). Matter contains the atoms, protons, neutrons and electrons that define the basic structure of each safety hazard. Each safety hazard has the following structural characteristics.

### All safety hazards possess tangibility (contactability or collision potential)

Safety hazards can make real contact with other safety hazards, and it is through this contact that safety hazards become dangerous. The matter base of all safety hazards forms the basis of their tangibility which is the basis of the threats that they offer or pose (Fields 2012:64) via their closing and collision potential (Van Fleet 2000:112).

### All safety hazards possess density (thickness or thinness)

Matter makes up the physical property of a safety hazard. Such physical properties come in different forms or states of density.

**FIGURE 1:**
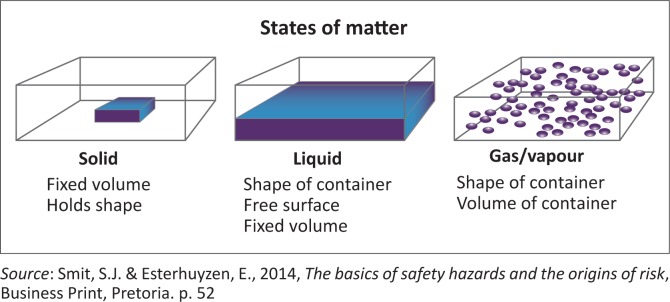
States of matter.

Density represents the range of thickness or thinness (solidness) of the safety hazards (Ophardt 2003:1). The density of a safety hazard (substance) is determined by the compactness of the atoms of the matter base making up such a safety hazard. Basically, density occurs in three states, namely solids, liquids and gases or vapours, as illustrated in Figure 1.

The more compact the atoms, the more solid the safety hazard (substance) and the more such safety hazards are inclined to retain their form under normal living conditions (Hallowell, Alexander & Gambatese 2016:68). Liquids are more plastic than solids, their component parts may move freely within the substance, although they do not separate from the basic matter (Hallowell et al. 2016:68). Liquids are inclined to take the form of their containers. In relation to solids and liquids, the forces of gases and vapours are weak, and this forces them to take the shape and volume of their containers (Energy 2005:5). The atoms of a gas and vapour will disperse and spread as far as the container will allow. The density of a safety hazard provides the experience of tangibility via the human senses, and the density of a safety hazard is changeable.

### All safety hazards come in various sizes or volumes

The size, also implying volume, refers to the three-dimensional measures of tangible objects, such as length, breadth and height or depth. Size is indicative of how much space or volume a safety hazard occupies. In terms of the three dimensions of measure, size is defined as big or small. Volume indicates a spherical shape and is measured in terms of diameter or radius in different ratios (Fleming & Fischer 2017:58). The size and volume do not determine the density of a safety hazard, while density does not determine size or volume.

### All safety hazards have weight or mass

All safety hazards have a matter base that determines the mass of each safety hazard and which forms the basis of the weight (Fleming & Fischer 2017:58). Weight relates to the mass and indicates the strength of the gravitational force that the mass of such a safety hazard exercises in a downward force towards the centre of the earth. Therefore, weight is a measure of gravitational force.

Mass and weight relate to density. The more mass a safety hazards has, and the more the atoms in the safety hazard are compacted, the denser and the heavier such a safety hazard will be.

### All safety hazards have shape

All safety hazards come in some sort of shape. Shape is depicted by the contour, outline or form of objects (i.e. safety hazards). Safety hazards come in different singular or a combination of geometrical shapes, such as round, flat, square, triangle, cube, tube, cylinder and many more. Although the shape of solids remains rigid, fluids and gases or vapours take the shape of their containers and the contours thereof are sometimes very difficult to determine. This specifically applies to safety hazards that cannot be seen or detected.

### All safety hazards have a surface and texture

Surface refers to the area of contact between two or more safety hazards. Texture refers to the nature of the surface of a safety hazard. The surface could range from extremely rough to immaculately smooth, as well as different variables of interlocking and rippling (Hallowell et al. 2016:66). Solids are usually tangible, while fluids and gases have smooth intangible surfaces that, in many cases, cannot be observed or experienced.

## Functioning characteristics of safety hazards

Besides the structural characteristics, all safety hazards also have some functioning characteristics. Functioning characteristics refer to the ways safety hazards perform when they make contact with other safety hazards. Each safety hazard functions on a unique individualistic basis. The differences in the functioning of safety hazards relate to the differences in the nature of safety hazards. For example, water and air function differently from each other. All safety hazards possess the functioning characteristics as discussed below.

### All safety hazards function because of energy

Safety hazards are sources of energy (Grimaldi & Simonds (1989:181). All safety hazards have a material (matter) base which comprises molecules that constitute the rotation of atoms around a central core. This rotation confirms the presence of kinetic energy. The concept of energy that is fundamental to physical science implies the capacity to do work, for whatever purpose (Crowell 2006:45; S.A. Government 1993: Section 1). Energy is a prerequisite for any act or action to occur and can be regarded as the strength and vitality to initiate and prolong any activity or action.

Safety hazards (tangible substances) possess two basic forms of energy, namely kinetic and potential energy (Crowell 2006:68):

- **Kinetic energy:** The energy embedded in the matter base is inseparable from all safety hazards. The ability to move from one point to another (closing potential) on its own account is also part of many safety hazards. Such kinetic energy that enables safety hazards to move on their own account is inherently part of such safety hazards.
- **Potential energy:** Potential energy explicates the ability to do work in the different forms of configurated energies. Such energies are not inherently part of safety hazards, but can be brought on via the energies of other safety hazards. Potential energies can be regarded as energies that are stored in safety hazards and come into operation when making contact with other safety hazards. This also applies to kinetic energy that is not an inherent energy, but a potential energy of many safety hazards that do not possess inherent kinetic energy to create movement on its own account, but which can be moved because of potential energy as a result of making contact with other safety hazards.

**FIGURE 2:**
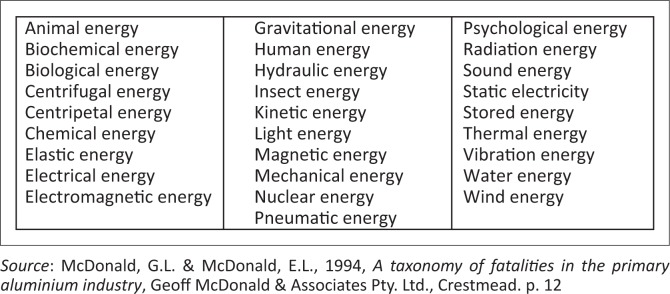
Basic manifestations and configurations of energies.

Examples of potential energies appear in Figure 2.

An example of the configuration of potential energy is striking a match on a match box that ignites a flame on the end of the match. Such flame produces thermal (heat) energy and light energy. The range of work that the energies of safety hazards can do is almost inexhaustible (Smit & Esterhuyzen 2014:67).

### All safety hazards function consistently

Safety hazards all have a matter base, and the nature of the matter base constitutes the functioning of the safety hazard in accordance with natural physical laws (Crowell 2006:72). To be governed by natural laws means that each safety hazard functions consistently in line with one or more specific natural law(s) that will apply to the specific safety hazard. For example, clear water will boil and evaporate if heated long enough and with sufficient thermal energy being presented by a microwave oven.

Natural physical laws show that they are (Crowell 2006:57):

- *true* by producing the same results under different circumstances in life
- *universal* by being active everywhere, irrespective of circumstances
- *simple* because the functioning and effects are easy to understand
- *absolute* because they remain consistently true to specific substances irrespective of circumstances
- *omnipotent*, in as far as all substances are bound by physical laws and function accordingly
- *conservative* because the nature of physical laws remains the same
- *consistent* because laws cannot be reversed over time.

As a result of consistency, the functioning of safety hazards can be predicted, such as water boiling when heated by an effective source of thermal energy. The consistent functioning of safety hazard results from natural symmetry. Symmetry refers to the prescription for safety hazards (substances) to function in accordance with natural physical laws. Such symmetry applies unconditionally to each unique safety hazard.

### All safety hazards function through interaction

Because of a matter base, all safety hazards are tangible, and safety hazards can make contact with other safety hazards. When safety hazards make contact, they interface or interact with one another. Such interaction occurs reciprocally. Safety hazard interacts through a process of energy exchange. Energy exchange implies energy being transferred between the safety hazards which are making contact. Such energy transfer has two-way effects on the safety hazards involved in the interaction. The exchange or transfer of energy happens between any part, particles or field of particles of any of the safety hazards involved in the interaction. The effect or outcome of the interaction and exchange of energies results in the change or state or condition of the safety hazards involved. For example, to boil water, a container with water and a source of heat (thermal energy) are needed. To heat up the water to boiling point requires a heating source transferring heat to the container which will transfer the heat to the water until it boils. The surrounding air will transfer cool air to the water, as will the water to the container, as will the container to the heat source. The effect of the exchange of energies between the safety hazards involved will be that the water eventually boils.

## Positioning the human being

At this point, a very basic and fundamental question arises. Where does the human being fit into the equation? The answer to such a question lies in determining whether the human meets the qualities of being a safety hazard and determining to what extent the human being reveals characteristics similar to safety hazards.

### Humans meeting the qualities of real safety hazards

In answering this question, it needs to be determined to what extent the nature of the human being is congruent with the basic qualities of real safety hazards.

From the data in Table 2, the following can be concluded:

- The human being is tangible.
- The human being possesses movability.
- The human being possesses interactability.
- The human being always stays unambiguous under all circumstances in life.

**TABLE 2:** The human being as a safety hazard.

Substance	Safety hazard qualities with confirmation of validity							
	Tangibility		Movability		Interactability		Unambiguousness	
	Yes	No	Yes	No	Yes	No	Yes	No
Human being	√	√	√	√	√	√	√	√

The data in the table confirm that the human being is fully congruent with the qualities of a safety hazard. Therefore, the human is a safety hazard and remains as such under all circumstances in life.

### Humans and safety hazard characteristics

The human is undoubtedly a safety hazard irrespective of the circumstances. This produces the question whether the human as a safety hazard also possesses the characteristics of a safety hazard? Details in Table 3 provide answers to this uncertainty.

**TABLE 3:** Congruency of the human as safety hazard with safety hazard characteristics.

Variable	Human being as safety hazard
**Safety hazard characteristics**	
Structural characteristics	
Yes	√
No	-
Tangibility	
Yes	√
No	-
Density	
Yes	√
No	-
Size	
Yes	√
No	-
Weight	
Yes	√
No	-
Shape	
Yes	√
No	-
Surface or texture	
Yes	√
No	-
Functioning characteristics	
Yes	√
No	-
Energy	
Yes	√
No	-
Consistency	
Yes	√
No	-
Interaction	
Yes	√
No	-
**Characteristic unique to humans**	
Inconsistency	
Yes	√
No	-

Details in the table above confirm congruency between the human being as a safety hazard and safety hazard characteristics. Such characteristics comprise both structural and functioning characteristics.

### Structural safety hazard characteristics of humans

Humans as safety hazards possess the following structural safety hazard characteristics (Smit & Esterhuyzen 2014:83–88):

- As a safety hazard, the human has a matter base, therefore the human has collision potential and therefore the human is tangible and contactable.
- As a safety hazard, the human has density in the body, life systems, water and gas, from conception through all the life stages (Perlman 2014:1).
- As a safety hazard, the human body comes in different sizes and diverse volumes as long as life prevails and thereafter.
- As a safety hazard, the human body presents in weight (mass) of different measures, for example, there is a difference in the weight of babies and Samurai wrestlers.
- As a safety hazard, the human presents in an extreme range of different shapes regarding, in general, overall body (morphology and obesity), head, hands, fingers, bellies, buttocks, feet, gender and more.
- The human has surface and texture as seen in terms of skin, hair, nails and more.

### Functioning safety hazard characteristics of humans

The following functioning safety hazard characteristics apply to the human as a safety hazard (Smit & Esterhuyzen 2014:88–92):

- As safety hazards, humans possess both inherent and potential energy, which presents in the many functions of the human body and life systems. The human can also can generate, utilise and control inherent energy, as well as numerous configurations of a range of energies.
- As safety hazards, humans function with consistency in terms of the natural laws that apply to almost all the systems of the human body and life processes. The consistent functioning of the human system occurs as a result of human genetics and the human’s adaptations to life circumstances (Tooby & Cosmides 1990:17).
- As safety hazards, humans possess the ability to interact with almost any other safety hazard based on the need to eat, to live, to relax, to work, to perform, to achieve and to excel.

### Humans function with inconsistency

The data in Table 3 show an additional functioning safety hazard characteristic, which only apply to the human being. Although the skeletal and biological systems of the human function on a consistent base in accordance with natural laws, the human mind functions on a different basis. The human mind is norm-based, and its operation is based on a system known as ‘ought-to’. No natural laws that typically determine the conduct of the human apply to the human mind. This means that the human has the opportunity or freedom to choose what conduct to perform or follow in a situation. Based on the freedom of choice, the human may select to function completely inconsistently with norms, rules and the ‘ought-to’ expectation. For example, a human driver in a motor vehicle is expected to stop at a stop sign according to a traffic rule, but humans are inclined to display a variety of stopping behaviour that demonstrates their inconsistency (Smit & Esterhuyzen 2014:96–98). In comparison with all other safety hazards that display nine safety hazard characteristics, the human presents with an additional safety hazard, namely, inconsistency.

The human bodily structure and organic functioning are symmetrically determined by natural laws; however, human behaviour is directed or governed by normativity or ‘ought-to-ness’ (Chappell 2004:1). Normativity relates to the concept of norms. Norms direct the acceptability of human conduct, setting guidelines regarding how humans should behave in specific and different circumstances (Cummings & Worley 2005:484). Norms present written and unwritten directives for human behaviour.

The inconsistency of the human as a safety hazard is an issue of great concern in occupational health and safety (OHS) because of its role and share in creating safety threats in numerous situations in life.

The main concern for human inconsistency is that humans do not consistently follow rules as expected to keep them safe in life-threatening circumstances.

Whatever the nature of the safety hazard, all safety hazards, including the human, have specific structural and functioning characteristics in common. Such characteristics are given in Table 4.

**TABLE 4:** Structural and functioning characteristics of safety hazards.

Structural characteristics	Functioning characteristics
Tangibility	Energy
Density	Consistency
Size or volume	Interaction
Weight or mass	Inconsistency (only humans)
Shape or form	-
Surface or texture	-

## Definition of safety hazards

Considering the preceding argumentation, it is clear that a safety hazard is any physical substance or object that can impact life, both positively and negatively, as a result of a range of safety hazard characteristics related to its unambiguous closing, collision and interacting qualities (Smit & Esterhuyzen 2014:40; Van Fleet 2000:112).

### Types of safety hazards

Safety hazards come in the following three different types:

#### Single safety hazards

Single hazards are objects or substances that comprise pure matter and do not exist in combination or mixture with any other matter. Examples of pure single safety hazards are iron, lead, oxygen, copper, hydrogen gold, nitrogen and many more. All of such substances pose safety hazards and are listed by using atomic numbers as elements on the natural scientific periodic table (Zumdahl & Zumdahl 2007:55–56). Examples of these elements are included in Table 5.

**TABLE 5:** Examples of natural elements listed on the periodic table.

Element	Symbol
Gold	Au
Oxygen	O
Lead	Pb
Nitrogen	N
Hydrogen	H

All the natural elements possess the qualities and characteristics of a safety hazard. They all are safety hazards and remain safety hazards on an unambiguous basis (Crowell 2006:86).

#### Compound safety hazards

Physical objects as safety hazards that have a matter base (Zumdahl & Zumdahl 2007:25, 55) frequently exist as a mixture in union or combination with other substances that are safety hazards. Compound safety hazards are substances combined or mixed with other substances. Compound safety hazards exist in a single feature as a separate entity. Compound safety hazards can comprise any mixture or combination of two or more single or compound safety hazards (Sadegh et al. 2018:5470). Some good examples of a compound safety hazard are water (which comprise hydrogen and oxygen), metal alloys, hairspray and bubble gum.

Compound safety hazards unambiguously meet the qualities and characteristics of safety hazards. Compound safety hazards possess the combined nature of the safety hazards of which they are comprised. The characteristics of compound safety hazards relate to both the nature of the single as well as the compound safety hazards. In this regard, water applies as an example, because it has the characteristics related to the density of oxygen and hydrogen that are inhalable, and water that is not inhalable but can dissolve some other elements and safety hazards, such as salt and sugar.

Compound safety hazards exist as single entities via a blend of single and other compound safety hazards. In general, compound safety hazards can be dismantled and separated into the different separate single or compound safety hazards that form a specific compound safety hazard.

#### Multiple safety hazards

A multiple safety hazard comprises a combination, mixture or group of single or compound safety hazards. Multiple safety hazards could exist in single- or multiple-entity format, but they have various structures that are formed by separate safety hazards. All multiple safety hazards unambiguously depict the basic qualities and characteristics of safety hazards. The various single and compound safety hazards that make a multiple safety hazard function in accordance with the natural laws that symmetrically apply to the characteristics of the safety hazards comprising the multiple hazards. Multiple safety hazards can, in most cases, be dismantled or broken down into the single and compound safety hazards that make specific multiple safety hazards.

Examples of multiple safety hazards are:

- a bunch of keys that comprise a group of compound safety hazards of different shapes made up of metals, plastic and rubber
- a palisade fence with different shapes comprising an alloy of metals and paint
- a vehicle comprising a range of safety hazards in different structures, shapes, weights, sizes, textures and many more
- the human being with all the different structural and functioning elements and characteristics.

#### Potential safety hazards

It has been extensively argued in this discussion that only substances that are made of matter, that are tangible, that can interact, that can move by itself or be moved and that unambiguously keep its basic characteristics are safety hazards. Should ‘anything’ not meet such criteria or requirements such a substance or object can never be a safety hazard.

A potential hazard implies that a substance or object in its current state is on the way to becoming a safety hazard and is developing a matter base. If such a substance or object does not have a matter base, it is intangible, and it cannot make contact with any other safety hazard. Such a substance or object cannot present a threat that can result in harm, damage or loss. The fact is that substances or objects with a matter base are safety hazards, and substances or objects that do not have a matter base will never have the potential to be or become safety hazards.

Therefore, there is no such thing as a potential safety hazard, as such things never show any signs of the qualities or characteristics of safety hazards. Table 6 depicts to what extent the types of safety hazards meet the qualities and characteristics of safety hazards.

**TABLE 6:** Types of safety hazards meeting safety hazard qualities and characteristics.

Types of safety hazards	Qualities and characteristics of safety hazards					
	Qualities of safety hazards			Characteristics of safety hazards		
	Some	All	None	Some	All	None
Single safety hazards	-	√	-	-	√	-
Compound safety hazards	-	√	-	-	√	-
Multiple safety hazards	-	√	-	-	√	-
Potential safety hazards	-	-	√	-	-	√

The data in Table 6 confirm that the basic types of safety hazards meet the qualities and characteristics of safety hazards, but that potential safety hazards are non-existent.

Potential safety hazards have no basis for consideration in the field of safety. However, what does need to be considered in the field of safety is the potential safety risks associated with every safety hazard.

### Ethical considerations

Ethical clearance was obtained from the Department of Business Management at the University of South Africa. (reference: 2015_CRERC_039[FA]).

## South African small business owner or manager cognisance of safety hazards

The *OHS Act*, No. 85 of 1993 as amended by the *OHS Act*, No. 181 of 1993 stipulates that it is the responsibility of employers to eliminate or mitigate all safety hazards before resorting to personal protective equipment (PPE). It should be reiterated that full compliance to such responsibility is legally required of all businesses, regardless of size or number of employees. The *OHS Act* does not make any distinction between small or large businesses regarding this responsibility (RSA 1993). With small businesses being the driving force of the South African economy, the necessity to determine if such legal responsibility is applied in small businesses necessitated the need to investigate compliance.

The small business owners or managers who participated in this study were required to indicate their actual behaviour related to the elimination or mitigation of all hazards before resorting to PPE on a scale of 1–3, with 1 equalling ‘do not comply at all’, 2 constituting ‘partially comply’ and 3 indicating ‘full compliance’. This research has proven that small business owners or managers do not fully comply with this responsibility, with an average score of 2.67 out of 3, with only 293 out of the 350 participants rating themselves. The remaining 57 participants either did not know if they complied or deemed compliance to this responsibility as not being applicable to them (Esterhuyzen 2017). Table 7 indicates the descriptive statistics regarding small business owner or manager responses with regard to the employer responsibility comprising the elimination or mitigation of all hazards before resorting to PPE.

**TABLE 7:** Descriptive statistics on actual behaviour of small business owners or managers regarding hazards in the workplace.

Employer responsibility in terms of the OHS Act	*n*	Mean	Median	Mode	Standard deviation	Skewness	Kurtosis
Eliminate or mitigate all safety hazards before resorting to PPE	293	2.67	3.00	3	0.57	−1.24	0.57

*Source*: Esterhuyzen, E., 2017, *Occupational health and safety: A compliance management framework for small businesses in South Africa*, University of South Africa Pretoria

OHS, occupational health and safety; PPE, personal protective equipment; *n*, number.

Even though the results do indicate ratings leaning more towards ‘full compliance’, the ratings also indicate that the 293 participants who did rate themselves only did so at an average score of 2.67. Such a score indicates that not all small business owners or managers perceived their businesses as being fully compliant in this regard, which might indicate ignorance of such a legal responsibility. The remaining 57 participants did not realise the importance regarding the elimination or mitigation of safety hazards in their respective workplaces before resorting to PPE (Esterhuyzen 2017). Therefore, small business owners should be educated on the fundamentals of safety hazards with a view of achieving complete legal compliance in this regard. It is also suggested that further research be conducted in this regard to enhance the small business owners’ or managers’ knowledge and understanding of safety hazards.

## Conclusion

The preceding arguments clearly declare that only substances that have a matter base and that are tangible have the potential to cause harm or damage. Anything that does not have a matter base, and which would not come in contact with other substances or objects cannot be regarded a safety hazard.

In addition, safety hazards need to unambiguously remain safety hazards under all circumstances in life, or else they are not safety hazards. The discussion clearly confirmed the validity that only objects with a matter base can remain safety hazards, irrespective of circumstances. Furthermore, it was argued that safety hazards have nine common characteristics that play a role in creating threats reciprocally to the well-being or safety of other safety hazards. It was also demonstrated that humans are safety hazards and that they possess one more of the nine common characteristics that all safety hazards do show.

An important element of the reasoning was the proof that none of the current definitions of safety hazards that appear in the literature really describe safety hazards, because they do not meet any of the scientific qualities and characteristics of safety hazards. The same argumentation confirmed the non-existence of potential safety hazards. The discussion finally presented an acceptable and valid definition of a safety hazard with proof that substances with a matter base are the only safety hazards that unambiguously remain safety hazards at all times under all circumstances.

Employers, such as small business owners or managers, should thus ensure that safety hazards are identified and addressed based on the scientific perspective of safety hazards. Only when safety hazards are correctly identified, can proper procedures be put in place to eliminate or mitigate such safety hazards, with a view of ensuring the safety and well-being of employees. This research has indicated that not all small business owners or managers are compliant with this legal responsibility, and that assistance should be provided to small business owners or managers to assist them in realising the importance of safety hazards in the workplace. Proper cognisance of safety hazards leads to enhanced compliance with legislative requirements.
